# Advanced Nanomaterials in Biomedical Application

**DOI:** 10.3390/nano13101625

**Published:** 2023-05-12

**Authors:** Goran N. Kaluđerović, Nebojša Đ. Pantelić

**Affiliations:** 1 Department of Engineering and Natural Sciences, University of Applied Sciences Merseburg, Eberhard-Leibnitz-Str. 2, 06217 Merseburg, Germany; 2 Department of Chemistry and Biochemistry, Faculty of Agriculture, University of Serbia, 11080 Belgrade, Serbia

Over the last few decades, great efforts have been dedicated to the discovery of various nanomaterials. Due to their unique optical, magnetic, and electrical properties (among others), they have found applications in medicine (drug delivery), agriculture, electronics, catalysis, etc. Thus, due to the increasing possibilities, the need to design and fabricate novel nanoparticles is rapidly increasing. In this Special Issue “Advanced Nanomaterials in Biomedical Application”, a total of seventeen articles—including five reviews—have been published, addressing the most recent advances in nanomaterials in terms of both synthesis and characterization as well as technological applications. In the following, we provide a brief overview of the key findings presented in this Special Issue.

In recent decades, platinum-based drugs have been widely used for the treatment of many types of cancer. However, their clinical utility is limited by severe side effects, such as neuro-, nephro-, and ototoxicity, and the development of resistance. One of the strategies for overcoming these issues is the development of nanoparticles that can enhance cellular accumulation in target cells and reduce the associated toxicity of drugs in normal cells. In this regard, Giusto et al. [1] prepared a 2D graphene-oxide-based nanoplatform functionalized with highly branched, eight-arm polyethylene-glycol, which enhanced the efficiency and loading capacity of platinum-based drugs, achieving a high-performance and stable nanodelivery system. The results have shown that the fabricated nanocarrier enables the application of lower amounts of Pt-drugs than a Pt-free complex to attain similar outcomes. Furthermore, the nanoplatform achieves excellent cellular proliferation inhibition in osteosarcoma, which is also observed in glioblastoma but in a less pronounced manner. Moreover, the presented nanoplatform also shows promise for inhibiting migration, especially in highly invasive breast carcinoma (e.g., MDA-MB-231 cells). Accordingly, the prepared nanoplatform represents an interesting tool for the treatment of various cancers. In addition, Predarska and co-workers [2] reported the synthesis of three novel platinum(IV) conjugates of cisplatin, containing derivatives of caffeic and ferulic acid in their axial positions, and their immobilization into SBA-15 particles for the preparation of corresponding mesoporous silica nanoparticles (MSNs). The prepared complexes showed higher or comparable antiproliferative activity with respect to cisplatin against four human breast cancer cells (BT-474, MCF-7, MDA-MB-468, and HCC1937). This activity increased significantly after immobilization in SBA-15, and the IC_50_ values were more than 1000 times lower compared to cisplatin. Furthermore, the derivative with the highest activity cisplatin–diacetyl caffeate conjugate and its MSNs induced apoptotic cell death by causing potent caspase activation. Moreover, in vivo studies conducted using BALB/c mouse models with breast tumors showed that the same compound and its MSNs exhibit tumor growth inhibition with a reduced necrotic area and lowered mitotic rate. The review paper by Spoială [3] focused on smart magnetic drug delivery systems for cancer treatment. The authors comprehensively describe the effectiveness of using nanotechnology and magnetic nanoparticles to facilitate the early detection and selective destruction of cancer cells. This review will help new researchers to obtain comprehensive information in the field of drug delivery systems for cancer diagnosis and treatment. Peserico et al., described the usefulness of nanoparticles (NPs) in the diagnostic and/or therapeutic sector [4]. The review presents a comprehensive summary of the accessible technologies targeting cell–NP interaction and/or detection in cancer and regenerative medicine. The key nanocarrier-impacting elements (e.g., the typology and functionalization of NPs, the tuning capacity of cells’ in vitro and in vivo interaction mechanisms, and labeling with NPs) were analyzed.

The rapid spread of bacteria and antibiotic resistance requires new infection control strategies. In this sense, nanomaterials seem to be promising tools for maximizing drug activity due to their unique size and properties. Quach and co-authors [5] developed an efficient method for improving the antibacterial properties of graphene oxide (GO) by growing nanosilica (NS) on the surface of GO. The silver nanoparticles (AgNPs) that were immobilized on nanosilica to create a composite GO/NS/AgNPs exhibited remarkable antibacterial activity against *Escherichia coli* and *Bacillus subtilis*, suggesting that this system has great potential as an efficient antibacterial coating for medical equipment and other surfaces. In the paper submitted by Lima et al. [6], a new nanoparticle-based approach to modulating the harmful inflammatory consequences of fungal infection for the host using β1,3-glucan-functionalized polystyrene nanoparticles (β-Glc-PS) was presented. Moreover, these nanoparticles were able to down-modulate a *Candida-albicans*-induced proinflammatory response of host immune cells in a size-dependent manner. In the study by Salmerón-Valdés et al. [7], the antibacterial activity against *Streptococcus mutans* and the mechanical properties of conventional and hybrid type I glass ionomers modified with and without halloysite nanotubes loaded with chlorhexidine were investigated. Based on the obtained results, the authors concluded that the addition of nanotubes preloaded with chlorhexidine at concentrations of 5% and 10% effectively inhibited the presence of *S. mutans*, especially regarding the dose–response relationship, while maintaining and improving mechanical properties. This suggests that the addition of halloysite nanotubes to conventional and resin-modified glass ionomer cements could be a new method for counteracting orthodontic ligament injury, offering the advantage of maintaining and improving mechanical properties.

The studies conducted by Xue et al. [8] have shown an opposite color response of a giant polyoxometalate, namely, a brown Keplerate cluster abbreviated as [Mo_132_] and containing 72 Mo(VI) and 60 Mo(V), to the existing states of the human papillomavirus (HPV) major capsid protein, L1-pentamer (L1-p), and virus-like particles (VLPs). The color responses result from the different binding modes between [Mo_132_] and the capsid protein. This straightforward colorimetry approach is of importance to estimating the existing states of the HPV capsid protein and could be used in the future to analyze the quality of the HPV vaccine and the existing states of other viruses.

Many studies have shown that CSC chemokine receptor 4 (CXCR4) is a promising target for cancer therapies, and intracellular siRNA delivery to suppress CXCR4 expression in cancer cells is an effective therapeutic strategy. Thus, Cao et al. [9] synthesized carriers, by preparing heptafluorobutyryl-polyethylene glycol-polyethyleneimine (FPP) used to coat magnetic nanoparticles (MNPs) to obtain magnetic nanocarriers, FPP@MNPs, for siRNA delivery and CXCR4 knockdown. The results show that the cellular uptake efficiency of the FPP@MNPs was significantly improved and that they exhibited low cytotoxicity. Furthermore, the siRNA transfection efficiency was validated in various cell lines, with the result showing that the developed nanocarriers could effectively reduce CXCR4 expression on the cell membrane.

Min and co-authors [10] presented a new type of injectable composite hydrogel using glycol chitosan (GCH), a water-soluble derivative of chitosan, and amino-functionalized bioactive glass nanoparticles (ABG NPs) to construct a single crosslinker using genipin (GN) or dual crosslinkers as a combination of GN and poly(ethylene glycol)diglycidyl ether (PEGDE). Using ABG NPs with GCH while employing GN as a single crosslinker, the fabricated ABG/GCH gels exhibited strength and elasticity that was moderately dependent on the spacer length of the ABG NPs. On the other hand, the combination of GN and PEGDE as dual crosslinkers significantly improved the strength and elasticity of the gels, while the gelation time was adjustable. Moreover, some optimally dual-crosslinked ABG/GCH gels were able to support the growth of seeded osteoblast-like cells and enhance matrix deposition. The obtained results indicate that a novel dual-crosslinked hydrogel has potential applications in bone repair.

The work of Tyubaeva et al. [11] explores the effect of hemin on the structure and properties of electrospun nanocomposite materials based on poly-3-hydroxybutyrate (PHB) and on their biocompatibility and antimicrobial activity. The results indicate that the presence of hemin significantly improves the structural properties of the material. The antimicrobial activity of hemin ensured that both Gram-negative and Gram-positive cultures died after contact with the PHB–Hmi fiber materials. These materials could be used for regenerative medicine, as dressing layers, as hygienic agents, as filter materials, and for other clinical products that require an advanced surface combined with antimicrobial properties and biocompatibility.

Ren and coauthors [12] summarized the recent advances in developing lipid-, metal-, carbon-, and polymer-based nanomaterials for antibacterial applications, covering the latest nanotechnologies for the design and development of nano- and nanocomposite materials designed to combat multidrug-resistant bacteria. Moreover, further development of antimicrobial nanomaterials was discussed.

Ding et al. [13] designed protein corona cationic liposomes (CLs) with AT-1002 (TJ regulatory peptide) possessing a core–shell structure based on the characteristics of BSA. Liraglutide was effectively encapsulated in the CLs, with the drug EE% of the liposomes equaling 85 ± 5% and the average particle size equal to 203 ± 13 nm. The drug itself, liraglutide, showed good structural stability. The protein corona liposomes had a good intestinal internalization effect, longer intestinal absorption time, and good biological safety. The adaptive protein corona liposomes have potential applications in the oral administration of proteins and peptides.

In their review article addressing SARS-CoV-2, a hot topic lately, Kianpout et al. [14] gave an overview of the structure of the virus and the cause of its pathogenicity. Moreover, the authors examine the biotechnological methods for vaccine production and the available nano-based concepts for overcoming this viral pandemic. The optimal conditions for the production of nano-mediated vaccines are discussed, and biotechnological solutions for forthcoming viral strikes are examined.

A new nano-emulsion adjuvant based on squalane (SNA: Span85, Tween60, squalane, polyethene glycol-400) containing CpG was prepared, and its in vivo properties were examined [15]. The SNA particles (diameter ca. 95 nm) showed good stability and biocompatibility. SNA was used for the preparation of a foot-and-mouth disease virus-like particle vaccine. Within 4 weeks in BALB/c mice, the SNA-VLPs vaccine significantly increased specific antibody levels, including IgG1 and IgG2a and, in the immune serum, IFN-γ and IL-1β. In guinea pigs, upon treatment with SNA, a noticeable enhancement of specific and neutralizing antibodies was observed within 4 weeks, thus enabling the proliferation of splenic lymphocytes. Outstandingly, one dose of SNA-VLPs immunized the guinea pigs with a protection rate of up to 83%, which was comparable to the group treated with the ISA-206, indicating that this novel formulation is an effective adjuvant for the FMD-VLP vaccine.

Harada et al., reported a useful technique for protein delivery to macrophages [16]. By applying low-binding mutant SubAB_S35A_ (subtilase cytotoxin; S35A –in B subunit 35th serine mutated to alanine) with poly(d,l-lactide-co-glycolic) acid (PLGA) nanoparticles, the selective delivery of cytotoxin to macrophages, in comparison to epithelial cells, was suggested. This drug delivery system presents anti-inflammatory effects.

In the study by Wen et al. [17], osteoimmunomodulatory nanoparticles for bone regeneration, which is a complex process that involves osteoblasts and osteoclasts (skeletal cells) as well as immune cells, an overview of the most important literature is presented. Namely, the authors highlighted the importance of osteoimmunology in bone regeneration. Moreover, the progress and application of nanoparticle-based methods for bone regeneration and macrophage-targeting drugs, respectively, for advanced osteoimmunomodulation are summarized.
